# Prediction Models for Post‐Stroke Hospital Readmission: A Systematic Review

**DOI:** 10.1111/phn.13441

**Published:** 2024-10-14

**Authors:** Yijun Mao, Qiang Liu, Hui Fan, Erqing Li, Wenjing He, Xueqian Ouyang, Xiaojuan Wang, Li Qiu, Huanni Dong

**Affiliations:** ^1^ Catheterization Laboratory, Xianyang Central Hospital Xianyang City Shaanxi Province China; ^2^ Orthopedic Surgery, Xianyang Central Hospital Xianyang City Shaanxi Province China; ^3^ Nursing Department, Xianyang Central Hospital Xianyang City Shaanxi Province China

**Keywords:** evidence‐based nursing, prediction model, prognosis, readmission, risk assessment, stroke, systematic review

## Abstract

**Objective:**

This study aims to evaluate the predictive performance and methodological quality of post‐stroke readmission prediction models, identify key predictors associated with readmission, and provide guidance for selecting appropriate risk assessment tools.

**Methods:**

A comprehensive literature search was conducted from inception to February 1, 2024. Two independent researchers screened the literature and extracted relevant data using the CHARMS checklist.

**Results:**

Eleven studies and 16 prediction models were included, with sample sizes ranging from 108 to 803,124 cases and outcome event incidences between 5.2% and 50.0%. The four most frequently included predictors in the models were length of stay, hypertension, age, and functional disability. Twelve models reported an area under the curve (AUC) ranging from 0.520 to 0.940, and five models provided calibration metrics. Only one model included both internal and external validation, while six models had internal validation. Eleven studies were found to have a high risk of bias (ROB), predominantly in the area of data analysis.

**Conclusion:**

This systematic review included 16 readmission prediction models for stroke, which generally exhibited good predictive performance and can effectively identify high‐risk patients likely to be readmitted. However, the generalizability of these models remains uncertain due to methodological limitations. Rather than developing new readmission prediction models for stroke, the focus should shift toward external validation and the iterative adaptation of existing models. These models should be tailored to local settings, extended with new predictors if necessary, and presented in an interactive graphical user interface.

**Trial Registration:**

PROSPERO registration number CRD42023466801

**Abbreviations**
ADL: activities of daily living
ANN: artificial neural network
AR: all‐cause readmission
AUROC: area under the receiver operating characteristic curve
BBS: Berg Balance Scale
BI: Barthel Index
CBM: China Biology Medicine Disc
CC: case control
CI: confidence interval
CINAHL: cumulative index to nursing and allied health literature
CNKI: China National Knowledge Infrastructure
COX: Cox regression
CS: cross sectional
EMR: electronic medical record
EPV: events per variable
EQ5D: EuroQoL five‐dimensional questionnaire
FIM: function independent measurement
FOIS: Functional Oral Intake Scale
GBM: gradient boosting machines
IADL: instrumental activities of daily living
MMSE: mini‐mental state examination
KNN: k nearest neighbor
LDL: low‐density lipoprotein
LR: logistic regression
MBDETRC: medical big data engineering & technology research center
NBC: naive Bayes classifier
NHI: National Health Insurance Research Database
NIHSS: National Institute of Health Stroke Scale
NPAC: National Post‐Acute Care Database
NR: not reported
OR: odds ratio
PC: prospective cohort
PROBAST: prediction model risk of bias assessment tool
RC: retrospective cohort
RF: random forest
RHDS: Readiness for Hospital Discharge Scale
ROB: risk of bias
SR: stroke‐related readmission
SVM: support vector machine
TIA: transient ischemic attacks
UDSMR: uniform data system for medical rehabilitation
VIP: China science and technology journal database
XGBoost: extreme gradient boosting

## 1 Introduction

Stroke, a severe condition leading to focal neurological impairments, is the second leading cause of mortality and disability worldwide and the foremost contributor to both among adults in China (Correction to: An Updated Definition of Stroke for the 21st Century: A Statement for Healthcare Professionals from the American Heart Association/American Stroke Association; Hilkens et al., 2024; Wu et al. 2019), characterized by high incidence, disability, mortality, and recurrence rates (Group Report On Stroke Prevention 2023; Mi et al. 2023). Moreover, the incidence of stroke continues to rise annually. Stroke not only results in various functional impairments, such as motor, language, swallowing, and cognitive deficits (Lewsey et al. 2015) but also garners significant clinical attention due to its high recurrence and rehospitalization rates. The 30‐day readmission rate among stroke survivors ranges from 12.1% to 28.8%, with approximately 50% of these cases being preventable (Kumar et al. 2022; Qiu et al. 2021; Strowd et al. 2015; Wen et al. 2018). The global rate of unplanned readmissions among stroke patients has been increasing, largely due to infections, coronary heart disease, and stroke recurrence.

Readmission serves as a key indicator for evaluating healthcare quality and efficiency (El et al. 2024). Reducing unnecessary readmissions is crucial for quality improvement, as they have detrimental effects on both patients and healthcare systems. High readmission rates may indicate unresolved issues at discharge, suboptimal post‐discharge care, or the presence of multiple comorbidities. These rates impose a substantial economic burden on healthcare systems, while also presenting opportunities to reduce treatment costs and alleviate the financial strain on the healthcare system. Consequently, lowering readmission rates has become a critical objective in China's healthcare reform.

Stroke readmissions contribute to poor treatment outcomes, increased disability rates, and worsened long‐term prognosis, while significantly increasing treatment costs and straining clinical resources (Qiu et al. 2021). Given the clinical and policy significance of stroke, identifying readmission risk factors, accurately predicting readmission risks, and implementing preventive measures for patients most likely to benefit is essential for optimizing the allocation of scarce intervention resources. This approach assists clinicians and healthcare institutions in caring for stroke patients and reducing avoidable hospitalizations.

Stroke readmission rates are widely recognized as essential indicators for evaluating treatment outcomes and monitoring disease progression, both domestically and internationally (Liu et al. 2024). Controlling stroke readmission rates has become a focal point in clinical practice across various medical centers. Data‐driven clinical decision support tools, such as predictive models, offer clinicians precise predictions by analyzing past cases, potentially reducing the influence of cognitive biases. Developing efficient and scalable predictive models can help healthcare providers effectively identify high‐risk patients and implement preventive measures, ultimately reducing economic costs for both patients and the healthcare system.

Although numerous studies on predictive models for stroke readmissions exist, comprehensive systematic reviews assessing the methodological quality, predictive accuracy, and clinical applicability of these models are lacking. The objectives of this study are to (1) assess the methodological rigor and predictive performance, including discrimination and calibration, of existing models; (2) evaluate potential bias risks and clinical applicability of these models; (3) summarize and analyze predictive factors associated with stroke readmissions, identifying those that enhance prediction accuracy; and (4) understand the characteristics of high‐performing, scalable, and transparent predictive models.

## 2 Methods

### 2.1 Data Sources and Searches

We conducted a comprehensive search of the PubMed, Web of Science, Embase, cumulative index to nursing and allied health literature (CINAHL), China National Knowledge Infrastructure (CNKI), Wanfang Database, China Science and Technology Journal Database (VIP), and China Biology Medicine Disc (CBM) databases from their inception through February 2023. The search focused on articles related to readmission risk prediction models in stroke survivors. The search terms included three main concepts: stroke, readmission, and prediction.

### 2.2 Study Selection

Two reviewers independently screened the articles based on the inclusion criteria, with a third reviewer providing input in cases of disagreement. The inclusion criteria were (1) studies involving patients with stroke; (2) an observational study design; (3) an outcome of interest focused on readmission within 1 year of discharge; and (4) the reporting of a prediction model. The exclusion criteria were (1) studies addressing only risk factors for stroke readmission without constructing predictive models; (2) studies where the full text was not accessible; (3) gray literature, including conference abstracts and agency reports; (4) duplicate publications; and (5) studies not written in English or Chinese.

### 2.3 Data Extraction

Data extraction included information such as the first author, year of publication, data source, outcomes, sample size, handling of missing data, methods of derivation and validation, and predictors.

### 2.4 Assessment of Risk of Bias (ROB)

The ROB and applicability of the included studies were evaluated using the prediction model risk of bias assessment tool (PROBAST) (Moons et al. 2019).

## 3 Results

**FIGURE 1:**
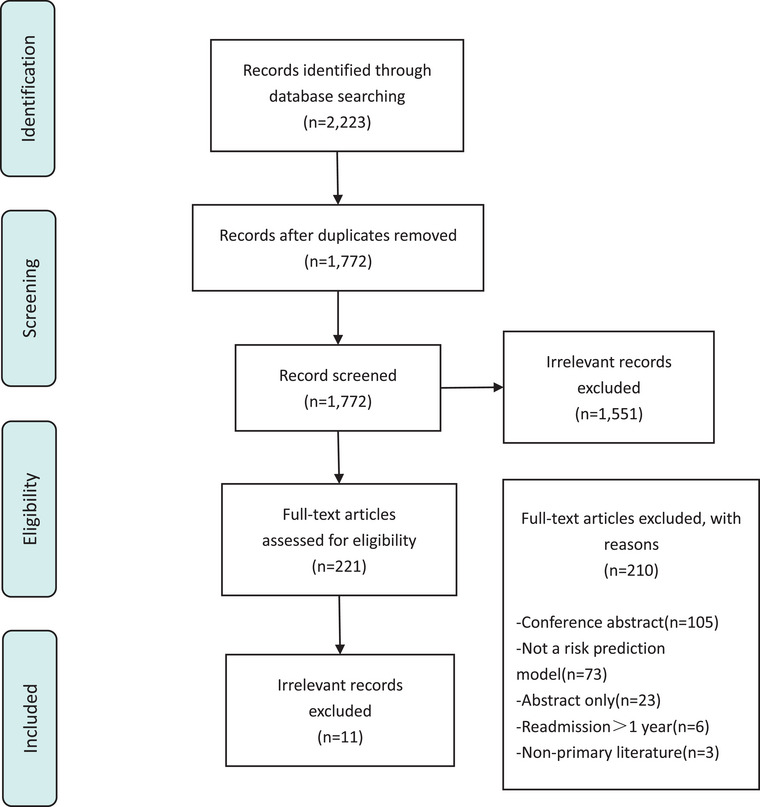
Search results and study selection. [Colour figure can be viewed at wileyonlinelibrary.com]

A total of 2223 records were identified through database searches, with 451 duplicates removed. After screening 1772 articles, 1551 irrelevant records were excluded. An additional 210 articles were excluded for the following reasons: conference abstract (*n* = 105), no risk prediction model (*n* = 73), abstract only (*n* = 23), readmission period exceeding 1 year (*n* = 6), and not primary literature (*n* = 3). Ultimately, 11 studies were included in the final analysis, reporting 16 prediction models for readmission in stroke survivors (Figure 1) (Chen et al. 2022; Darabi et al. 2021; Fehnel et al. 2015; Gao, Yan, and Lin 2022; Lineback et al. 2021; Miao 2022; Slocum et al. 2015; Sun et al. 2020; Tseng and Lin 2009; Wang 2021; Xu et al. 2019).

### 3.1 Characteristics of the Included Studies

Eleven studies (Chen et al. 2022; Darabi et al. 2021; Fehnel et al. 2015; Gao, Yan, and Lin 2022; Lineback et al. 2021; Miao 2022; Slocum et al. 2015; Sun et al. 2020; Tseng and Lin 2009; Wang 2021; Xu et al. 2019) were published between 2009 and 2022, with eight (Chen et al. 2022; Darabi et al. 2021; Gao, Yan, and Lin 2022; Lineback et al. 2021; Miao 2022; Sun et al. 2020; Wang 2021) published in the last 5 years. The outcomes of interest included all‐cause readmission (AR) (*n* = 7) (Chen et al. 2022; Darabi et al. 2021; Fehnel et al. 2015; Lineback et al. 2021; Slocum et al. 2015; Sun et al. 2020; Tseng and Lin 2009) and stroke‐related readmission (SR) (*n* = 5) (Gao, Yan, and Lin 2022; Lineback et al. 2021; Miao 2022; Wang 2021; Xu et al. 2019), with two studies focusing specifically on ischemic SR (*n* = 2) (Miao 2022; Xu et al. 2019). The follow‐up periods for readmission varied: 1 month (*n* = 9) (Chen et al. 2022; Darabi et al. 2021; Fehnel et al. 2015; Gao, Yan, and Lin 2022; Lineback et al. 2021; Miao 2022; Slocum et al. 2015; Sun et al. 2020; Wang 2021), 3 months (*n* = 1) (Xu et al. 2019), and both 3 months and 1 year (*n* = 1) (Tseng and Lin 2009). The reported readmission rates ranged from 5.2% to 50.0%. Three studies (Darabi et al. 2021; Miao 2022; Tseng and Lin 2009) reported methods for handling missing data: one study (Darabi et al. 2021) used multiple imputation, one study (Miao 2022) excluded variables with more than 20% missing date and used averages to replace missing data of 20% or less, and one study (Tseng and Lin 2009) directly deleted missing data (Table 1).

**TABLE 1:** **Basic characteristics of the included studies**.

						Outcome			Sample size	
Study	Country	Data source (study period)	Population	Age	Study design	Type of readmission	Length of time	Readmission rate (%)	Development	Validation
Tseng 2009	China	NHI (2000–2001)	Ischemic, hemorrhagic, TIA, or ill‐defined	≥ 18	RC	AR	90 d 1 y	28.6 50.0	468	NR
Fehnel 2015	US	NPAC (2008)	Ischemic	> 65	PC	AR	30 d	21.0	39,178	NR
Slocum 2015	US	UDSMR (2002–2011)	Ischemic or hemorrhagic	69.78 ± 13	CS	AR	30 d	10.6	803,124	NR
Xu 2019	China	MBDETRC (2017)	Ischemic	> 18	RC	SR	90 d	9.6	1214	4856
Sun 2020	China	EMR (2018–2019)	Ischemic	≥ 60	PC	SR	31 d	15.9	328	NR
Darabi 2021	US	EMR (2015–2018)	Ischemic	≥ 18	PC	AR	30 d	9.4	2548	636
Wang 2021	China	EMR (2010–2019)	lacunar infarct	NR	RC	SR	30 d	15.1	185	94
Lineback 2021	US	EMR (2011–2016)	Ischemic or hemorrhagic	> 18	RC	AR SR	30 d	14.0 5.2	2305	550
Gao 2022	China	EMR (2019–2020)	Ischemic	≥ 60	CC	SR	31 d	24.1	108	NR
Miao 2022	China	EMR (2020–2021)	Ischemic	≥ 18	PC	SR	30 d	13.7	750	NR
Chen 2022	China	EMR (2014–2019)	Ischemic or hemorrhagic	65.5 ± 13.0	PC	AR	30 d	8.1	1033	443 (167^a^)

Abbreviations: AR, all‐cause readmission; CC, case control; CS, cross sectional; EMR, electronic medical record; MBDETRC, medical big data engineering & technology research center; NHI, National Health Insurance Research Database; NPAC, National Post‐Acute Care Database; NR, not reported; PC, prospective cohort; RC, retrospective cohort; SR, stroke‐related readmission; TIA, transient ischemic attacks, UDSMR, uniform data system for medical rehabilitation.

^a^ Another 167 patients for external validation.

### 3.2 Prediction Model Approaches and Performance

In terms of model development, 10 studies (Darabi et al. 2021; Fehnel et al. 2015; Gao, Yan, and Lin 2022; Miao 2022; Slocum et al. 2015; Sun et al. 2020; Tseng and Lin 2009; Wang 2021; Wu et al. 2019; Xu et al. 2019) employed logistic regression (LR). Among the studies utilizing traditional biostatistical methods, six (Fehnel et al. 2015; Gao, Yan, and Lin 2022; Miao 2022; Slocum et al. 2015; Sun et al. 2020; Tseng and Lin 2009; Wang 2021) recommended LR for model development. In studies utilizing machine learning methods, three (Darabi et al. 2021; Xu et al. 2019) recommended extreme gradient boosting (XGBoost) as the preferred approach. Other recommended methods for model development were more varied and less consistently applied across the studies (Table 2).

**TABLE 2:** Recommendation models of inclusion literature.

Study	LR	XGBoost	RF	GBM	SVM	NBC	ANN	KNN	COX
Tseng 2009	√								
Fehnel 2015	√								
Slocum 2015	√								
Xu 2019		√							
Sun 2020	√								
Darabi 2021		√							
Wang 2021	√								
Lineback 2021		√							
Gao 2022	√								
Miao 2022			√						
Chen 2022							√		

Abbreviations: ANN, artificial neural network; COX, Cox regression; GBM, gradient boosting machines; KNN, K nearest neighbor; LR, logistic regression; NBC, naive Bayes classifier; RF, random forest; SVM, support vector machine; XGBoost, extreme gradient boosting.

Nine studies (Chen et al. 2022; Darabi et al. 2021; Fehnel et al. 2015; Gao, Yan, and Lin 2022; Lineback et al. 2021; Miao 2022; Slocum et al. 2015; Sun et al. 2020; Xu et al. 2019) reported model discrimination using the *C* statistic, with values ranging from 0.520 to 0.955. Six models demonstrated strong discrimination (*C* statistic> 0.75), with the best‐performing model developed by Sun et al. (2020), which achieved a *C* statistic of 0.955. Five studies (Gao, Yan, and Lin 2022; Miao 2022; Sun et al. 2020; Tseng and Lin 2009; Wang 2021) reported calibration using the Hosmer–Lemeshow goodness‐of‐fit test, all of which indicated good calibration. Six studies (Darabi et al. 2021; Lineback et al. 2021; Miao 2022; Slocum et al. 2015; Wang 2021; Xu et al. 2019) were internally validated; of these, four studies (Chen et al. 2022; Darabi et al. 2021; Lineback et al. 2021; Slocum et al. 2015) used cross‐validation, two studies (Wang 2021; Wu et al. 2019) employed random splitting, and two studies (Lineback et al. 2021; Miao 2022) used bootstrapping for validation. One study (Chen et al. 2022) used temporal validation for external validation (Table 3).

**TABLE 3:** **Domains of predictors and performance of stroke readmission risk prediction models**.

			Validation method			
Study	Modeling methods	Methods to handle missing data value	Internal validation	External validation	Discrimination (*C* statistic)	Calibration
Tseng 2009	LR	Delete	—	None	—	Hosmer–Lemeshow
Fehnel 2015	LR	NR	—	None	0.650	—
Slocum 2015	LR	NR	Cross‐validation	None	0.682	—
Xu 2019	XGBoost, LR	NR	Cross‐validation	None	Model 1:0.792 (0.717, 0.762) Model 2:0.739 (0.764, 0.818)	—
Sun 2020	LR	NR	—	None	0.955	Hosmer–Lemeshow
Darabi 2021	LR, RF, GBM, XGBoost, SVM	Multiple Imputation	Cross‐validation	None	0.740 (0.640, 0.780)	—
Wang 2021	LR	NR	Cross‐validation	None	—	Calibration curve
Lineback 2021	LR, NBC, SVM, RF, GBM, XGBoost	NR	Cross‐validation, bootstrap	None	Model 1:0.640 (0.630, 0.650) Model 2:0.580 (0.570, 0.590) Model 3:0.620 (0.610, 0.630) Model 4:0.520 (0.510, 0.530)	—
Gao 2022	LR	NR	—	None	0.825	Hosmer–Lemeshow
Miao 2022	LR, SVM, RF	Use averages to replace the missing data	Bootstrap	None	0.90	Calibration curve
Chen 2022	ANN, KNN, SVM, NBC, RF, COX	NR	Cross‐validation	Temporal validation	Model 1:0.940 (0.910, 0.970) Model 2:0.890 (0.850, 0.930)	—

Abbreviations: ANN, artificial neural network; COX, Cox regression; GBM, gradient boosting machines; KNN, K nearest neighbor; LR, logistic regression; NBC, naive Bayes classifier; NR, not reported; RF, random forest; SVM, support vector machine; XGBoost, Extreme gradient boosting.

### 3.3 Meta‐Analysis of the Incidence of Readmission in Stroke Patients

**FIGURE 2:**
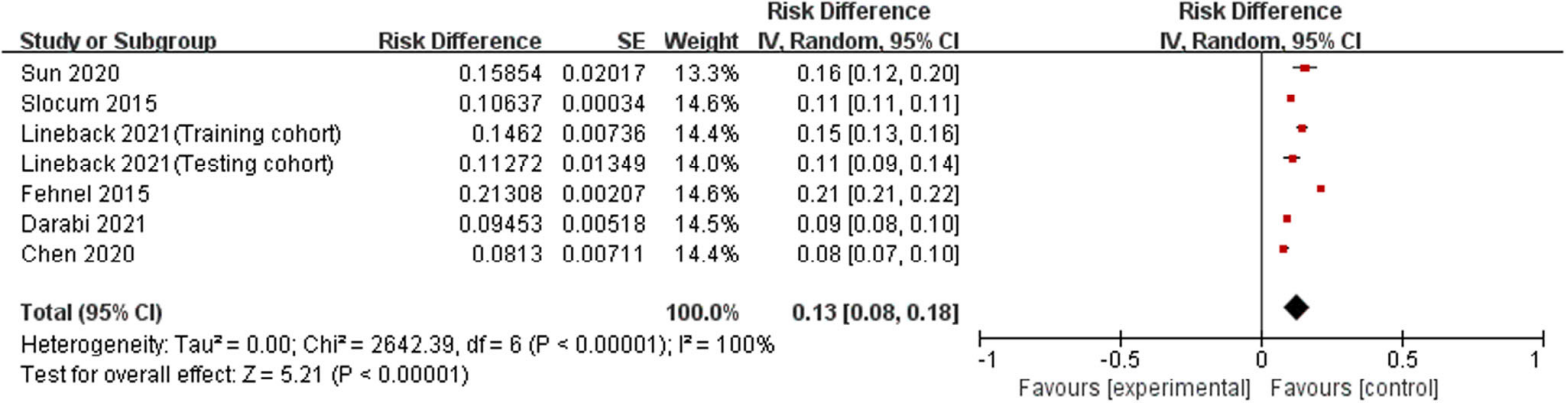
Forest plot of the incidence of AR in stroke patients. AR indicates all‐cause readmission; CI, confidence interval; SE, standard error. [Colour figure can be viewed at wileyonlinelibrary.com]

Six studies (Chen et al. 2022; Darabi et al. 2021; Fehnel et al. 2015; Lineback et al. 2021; Slocum et al. 2015; Sun et al. 2020) reported the incidence of AR in stroke patients, with significant heterogeneity across studies (*I* ^2^ = 100%, *p* < 0.001), which were analyzed using a random‐effects model. The meta‐analysis revealed that the overall incidence of AR in stroke patients was 13.0%, with a statistically significant difference (odds ratio [OR] = 0.13, 95% confidence interval [CI]: 0.08–0.18, *p* < 0.001) (Figure 2).

**FIGURE 3:**
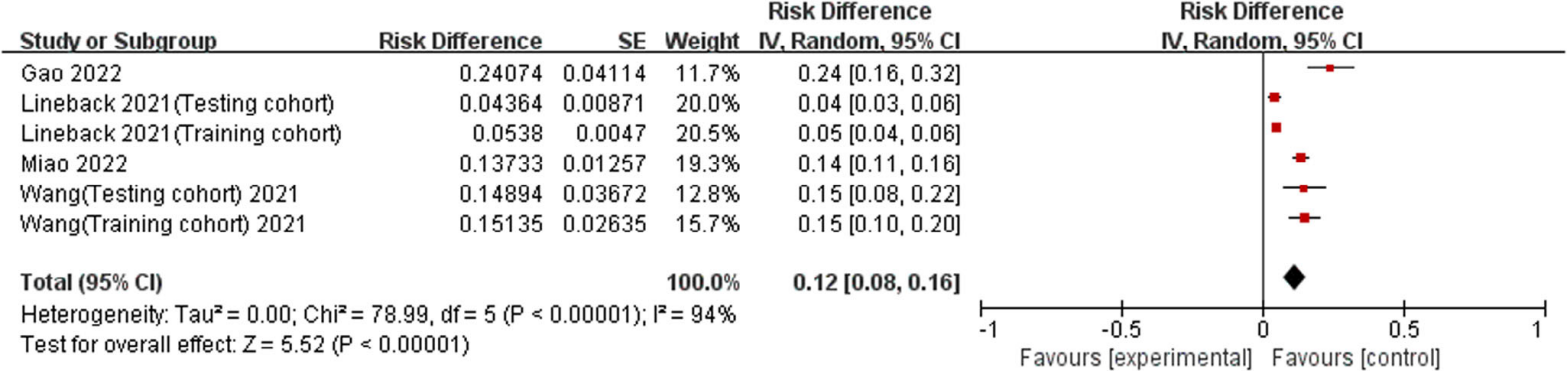
Forest plot of the incidence of SR in stroke patients. CI indicates confidence interval; SR, stroke‐related readmission. [Colour figure can be viewed at wileyonlinelibrary.com]

Four studies (Gao, Yan, and Lin 2022; Lineback et al. 2021; Miao 2022; Wang 2021) reported the incidence of SR, also showing significant heterogeneity (*I* ^2^ = 94%, *p* < 0.001), and were analyzed using a random‐effects model. The results indicated that the overall incidence of SR was 12.0%, with a statistically significant difference (OR = 0.12, 95% CI: 0.08–0.16, p < 0.001) (Figure 3).

### 3.4 Predictors of Readmission in Stroke Patients

The number of candidate predictors across the 16 different models ranged from 3 to 61. The most common predictors included length of stay (*n* = 5) (Gao, Yan, and Lin 2022; Miao 2022; Sun et al. 2020; Tseng and Lin 2009; Xu et al. 2019), hypertension (*n* = 4) (Gao, Yan, and Lin 2022; Miao 2022; Sun et al. 2020; Xu et al. 2019), age (*n* = 4) (Gao, Yan, and Lin 2022; Miao 2022; Slocum et al. 2015; Tseng and Lin 2009), and functional status (*n* = 4) (Chen et al. 2022; Miao 2022; Slocum et al. 2015; Sun et al. 2020) (Table 4).

**TABLE 4:** Predictors included in the model.

	Predictors				
	Type of readmission		Length of time		
Study	AR	SR	1 month	3 months	1 year
Tseng 2009	√				Age (per 5 years), length of stay(days), medical specialty of admission, hospital level
Fehnel 2015	√		Feeding tube, bowel incontinence, congestive heart failure, renal disease, chronic obstructive pulmonary disease		
Slocum 2015	√		Age, FIM rating, Charlson comorbidity index		
Xu 2019		√		Hypertension, red blood cell distribution width, direct bilirubin, length of hospital stay, pulmonary infection, alkaline phosphatase	
Sun 2020	√		Marital status, smoking, hypertension, hyperlipidemia, atrial fibrillation, ADL score, hospitalization days, RHDS score		
Darabi 2021	√		NIHSS > 24, insert indwelling urinary catheter, hypercoagulable state, percutaneous gastrostomy		
Wang 2021		√	Gender, blood glucose, hemoglobin		
Lineback 2021	√	√	—		
Gao 2022		√	Age, hypertension, LDL, length of stay		
Miao 2022		√	Smoking, hypertension, hyperlipidemia, hemoglobin a1c, high homocysteine		
Chen 2022	√		Post‐acute care, nasogastric tube, stroke type, BI score, IADL score, MMSE score, BBS score, FOIS score, EQ5D score		

Abbreviations: ADL, activities of daily living; AR, all‐cause readmission; BBS, Berg Balance Scale; BI, Barthel Index; EQ5D, EuroQoL five‐dimensional questionnaire; FIM, function independent measurement; FOIS, Functional Oral Intake Scale; IADL, instrumental activities of daily living; LDL, low‐density lipoprotein; MMSE, mini‐mental state examination; NIHSS, National Institute of Health Stroke Scale; RHDS, Readiness for Hospital Discharge Scale; SR, stroke‐related readmission.

### 3.5 ROB and Applicability Assessment

The “Participants” domain was assessed as having a high ROB in 54.5% of studies, primarily because retrospective designs may introduce information bias due to the unsystematic collection of predictor and outcome data, which is not recommended for prognostic modeling. Prospective cohort (PC) studies, which follow a longitudinal temporal logic of predictors and outcomes, are considered the optimal study design, as they capture disease progression in its natural state.

For the “Predictors” domain, 45.5% of studies were assessed as having a low ROB, particularly those that utilized PC designs where predictors were measured before outcomes occurred. However, 54.5% were assessed as having an unclear ROB because these studies did not disclose whether predictors were evaluated independently of outcome knowledge.

In the “Outcome” domain, 54.5% of studies were assessed as having a high ROB due to the retrospective design, where outcomes were determined before predictor measurements, potentially linking the outcome determination to the predictor. The remaining 45.5% were assessed as having an unclear ROB due to the lack of information on whether predictor data were clear at the time of outcome determination.

In the “Analysis” domain, 81.8% of studies were at high ROB. Six studies (Chen et al. 2022; Darabi et al. 2021; Gao, Yan, and Lin 2022; Miao 2022; Sun et al. 2020; Wang 2021) had insufficient sample sizes, failing to meet the criterion that the number of clinical outcome events should be 20 times greater than the number of potential predictors (Peduzzi et al. 1996). One study (Miao 2022) converted continuous variables into categorical ones, leading to potential information loss, abrupt changes in predictions near thresholds, reduced statistical efficacy, and decreased result credibility. Another study (Tseng and Lin 2009) directly deleted missing data, which may have introduced selection bias and resulted in information loss. Four studies (Chen et al. 2022; Gao, Yan, and Lin 2022; Sun et al. 2020; Tseng and Lin 2009) filtered predictors based on univariate analysis, potentially overemphasizing statistical significance while neglecting non‐significant variables that might still contribute to prediction.

**FIGURE 4:**
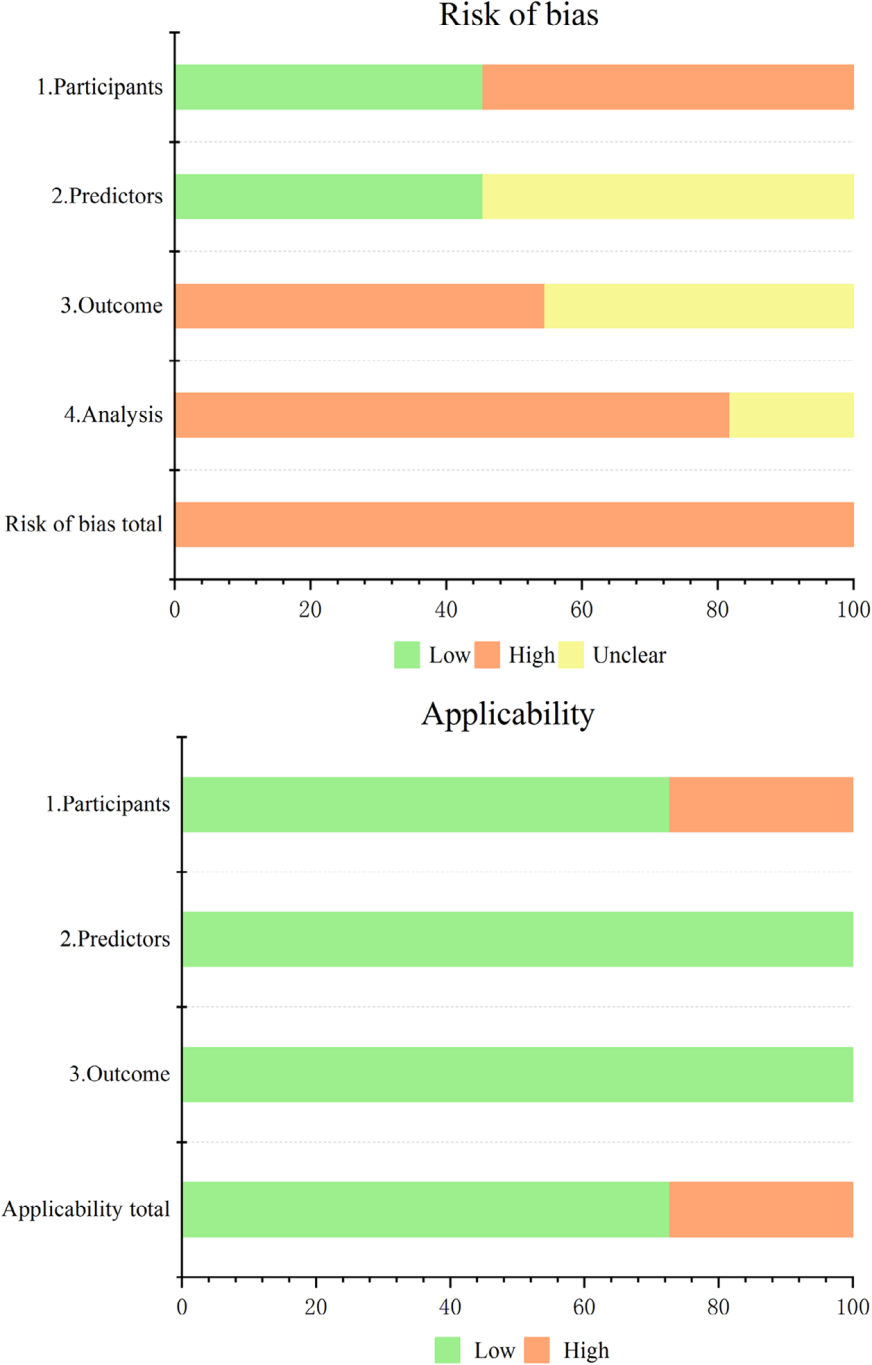
PROBAST risk of bias and applicability. PROBAST indicates the prediction model risk of a bias assessment tool. [Colour figure can be viewed at wileyonlinelibrary.com]

Regarding applicability, in the “Patients” domain, two studies (Gao, Yan, and Lin 2022; Sun et al. 2020) that included subjects aged ≥ 60 years and one study (Fehnel et al. 2015) that included subjects aged > 65 years were assessed as having high concerns about applicability. The “Predictors” and “Outcome” domains were assessed as having low concerns about applicability in all studies (Table 5, Figure 4).

**TABLE 5:** ROB appraisal results of eligible articles adapted from PROBAST.

	ROB				Applicability			Overall	
Study	Participants	Predictors	Outcome	Analysis	Participants	Predictors	Outcome	ROB	Applicability
Tseng 2009	−	？	−	−	+	+	+	−	+
Fehnel 2015	+	+	？	−	−	+	+	−	−
Slocum 2015	−	？	−	？	+	+	+	−	+
Xu 2019	−	？	−	−	+	+	+	−	+
Sun 2020	+	+	？	−	−	+	+	−	−
Darabi 2021	+	+	？	−	+	+	+	−	+
Wang 2021	−	？	−	−	+	+	+	−	+
Lineback 2021	−	？	−	？	+	+	+	−	+
Gao 2022	−	？	−	−	−	+	+	−	−
Miao 2022	+	+	？	−	+	+	+	−	+
Chen 2022	+	+	？	−	+	+	+	−	+

*Note*: ?, unclear; −, high risk of bias; +, Low risk of bias.

Abbreviations: PROBAST, prediction model risk of bias assessment tool; ROB, risk of bias.

## 4 Discussion

### 4.1 Model Performance and Quality Analysis of Study

Predictive models should exhibit both high discrimination and calibration, with discrimination being a prerequisite for ensuring good calibration (Mo et al. 2024). The area under the receiver operating characteristic curve (AUROC) for the 16 predictive models included in this study ranged from 0.520 to 0.955, with six models demonstrating an AUC greater than 0.75, indicating strong discrimination. Discrimination reflects the ranking of risk scores or predicted probabilities but does not indicate the accuracy of the model's risk predictions. Therefore, calibration is also necessary to quantitatively assess the model. Two studies (Gao, Yan, and Lin 2022; Wang 2021) used calibration curves to evaluate the model calibration, which is currently the best method for assessing the relationship between the model's predicted and actual observed values.

Models are typically presented through point score systems, graphical scales, nomograms, websites, and applications (Bonnett et al. 2019). Two studies (Miao 2022; Wang 2021) presented clinical prediction models using nomograms, while two others (Gao, Yan, and Lin 2022; Sun et al. 2020) used regression equations. In the future, it is recommended that risk predictions be calculated through web calculators or applications, transforming complex statistical models into interactive, user‐friendly interfaces accessible to healthcare professionals and patients.

The overall ROB in the prediction models was high, primarily due to the following reasons:

1. **Insufficient Sample Size**: The events per variable (EPV) principle is commonly used to estimate sample size in clinical prediction models, requiring at least 20 outcome events per predictor variable (Peduzzi et al. 1996). For example, if a model aims to include 10 predictors, at least 200 outcome events would be needed. A sufficient sample size ensures model performance, prevents overfitting, and improves reliability and generalizability (Wang and Wang 2023).
2. **Conversion of Continuous to Categorical Variables**: Converting continuous variables to categorical ones can reduce predictive power and lead to information loss. It is preferable to retain continuous variables, convert them to ordinal variables, or determine an optimal cutoff point using methods such as the log‐rank test of the Kaplan–Meier curve, the Youden index of the Receiver Operating Characteristic curve, or restricted cubic splines (Bennette and Vickers 2012).
3. **Handling of Missing Data**: Excluding subjects with missing data can compromise the objectivity of the sample and the efficacy of statistical results (Srijan and Rajagopalan 2024). It is recommended to use multiple imputations to fill in missing data, followed by sensitivity analysis to evaluate the stability of the results against a complete dataset or data analyzed using propensity score matching.
4. **Filtering Candidate Predictors Based on Univariate Analysis**: Relying solely on univariate analysis may overlook important variables, as statistical insignificance does not necessarily indicate that a predictor does not affect the outcome. Candidate predictors can be selected based on (a) previous literature reports; (b) medical knowledge and pathophysiological mechanisms; or (c) statistical analysis, comparing the efficacy of different predictive models to select the most appropriate predictors (Peduzzi et al. 1996).
5. **Lack of Calibration**: Six studies (Chen et al. 2022; Darabi et al. 2021; Fehnel et al. 2015; Lineback et al. 2021; Sun et al. 2020; Xu et al. 2019) did not report calibration, which is as critical as discrimination for assessing the consistency between predicted and observed risks. The best method to evaluate calibration is through calibration plots, which visually display the relationship between predicted probabilities and actual outcomes (Pate et al. 2024).
6. **Absence of Internal Validation**: Four studies (Fehnel et al. 2015; Gao, Yan, and Lin 2022; Sun et al. 2020; Tseng and Lin 2009) did not perform internal validation. Bootstrap validation is recommended when the dataset is small and it is challenging to effectively split the data into training and test sets. This method avoids the sample reduction caused by cross‐validation and does not require additional assumptions or new samples (Ecker et al. 2024).

### 4.2 The Predictors Used in Prediction Model

The number of predictors in the models across the 11 studies ranged from 3 to 11. The most frequently used predictors were length of stay (*n* = 5) (Gao, Yan, and Lin 2022; Miao 2022; Sun et al. 2020; Tseng and Lin 2009; Xu et al. 2019), hypertension (*n* = 4) (Gao, Yan, and Lin 2022; Miao 2022; Sun et al. 2020; Xu et al. 2019), age (*n* = 4) (Gao, Yan, and Lin 2022; Miao 2022; Slocum et al. 2015; Tseng and Lin 2009), and functional status (*n* = 4) (Chen et al. 2022; Miao 2022; Slocum et al. 2015; Sun et al. 2020). For stroke patients with critical conditions, surgical treatments, or slow recovery, the length of stay is often prolonged, which may increase the risk of readmission after discharge due to inadequate care and poor rehabilitation outcomes.

Elderly patients frequently present with hypertension and other comorbidities. Hypertension is a significant factor in the development of atherosclerosis, which greatly influences the likelihood of readmission in cases of cerebral infarction. Therefore, it is crucial to actively manage these underlying conditions and improve patient adherence to treatment and medication to control blood pressure, thereby reducing the risk of stroke recurrence. Functional status is another critical predictor, as it strongly influences the quality of life and rehabilitation outcomes for stroke patients. Those with better functional status tend to experience a higher quality of life and more successful rehabilitation, leading to a lower incidence of stroke recurrence or other related complications (Guan et al. 2017). These findings suggest that healthcare professionals should prioritize care for stroke patients with prolonged length of stay, hypertension, advanced age, and poor functional status.

### 4.3 Clinical Practice Recommendations

In recent years, artificial intelligence and machine learning have garnered increasing attention and are widely used in model development. Future research should consider combining traditional biostatistical methods with artificial intelligence or machine learning techniques to build clinical prediction models, potentially achieving better results than traditional methods alone. External validation was conducted in only one of the included studies; therefore, it is recommended that models undergo external validation through temporal, geographic, and domain‐specific validations. These validations should utilize data from different time periods, other centers or countries, and various clinical scenarios to assess the transferability and generalizability of the models.

Clear and precise presentation of clinical prediction models is essential to ensure that other researchers can independently validate them. In addition to providing the full model equations, it is suggested that web calculators or mobile applications embed these equations into a backend system that connects with healthcare electronic information systems. This integration would automate data entry and enhance usability.

## 5 Limitations

Despite conducting a thorough search, we may have missed studies published in languages other than English or Chinese. Additionally, gray literature, such as conference abstracts and agency reports, was excluded due to the lack of rigorous peer review.

## 6 Conclusion

This systematic review included 16 readmission prediction models for stroke, which generally exhibited good predictive performance and can effectively identify high‐risk patients likely to be readmitted. However, the generalizability of these models remains uncertain due to methodological limitations. Independent predictors of readmission risk in stroke patients include length of stay, hypertension, age, and functional status.

The complexity of disease management and the high rates of readmission among stroke patients underscore the need for innovative risk prediction models. Although there is a substantial number of readmission risk prediction models for stroke, many are of limited practical value due to the lack of external validation. Existing models require updating and validation before they can be reliably used in clinical practice. The absence of independent validation studies, high ROB, and low consistency in measured predictors limit the applicability of these models.

Rather than developing new readmission prediction models for stroke, the focus should shift toward external validation and the iterative adaptation of existing models. These models should be tailored to local settings, extended with new predictors if necessary, and presented in an interactive graphical user interface, such as websites and applications, that can be easily used by practitioners, policymakers, and guideline developers. This approach will facilitate the development of appropriate interventions for patients at different risk levels, promote the equitable distribution of medical resources, and ultimately reduce the readmission rates of stroke patients (Abreu et al. 2024).

## Data Availability

The data that support the findings of this study are available on request from the corresponding author. The data are not publicly available due to privacy or ethical restrictions.
